# Magnetic resonance imaging of mouse brain networks plasticity following motor learning

**DOI:** 10.1371/journal.pone.0216596

**Published:** 2019-05-08

**Authors:** Alexandra Badea, Kwan L. Ng, Robert J. Anderson, Jiangyang Zhang, Michael I. Miller, Richard J. O’Brien

**Affiliations:** 1 Center for In Vivo Microscopy, Department of Radiology, Duke University Medical Center, Durham, NC, United States of America; 2 Department of Neurology, Duke University Medical Center, Durham, NC, United States of America; 3 Brain Imaging and Analysis Center, Duke University, Durham, NC, United States of America; 4 Department of Neurology, UC Davis School of Medicine, Davis, CA, United States of America; 5 Bernard and Irene Schwartz Center for Biomedical Imaging, Department of Radiology, New York University School of Medicine, New York, NY, United States of America; 6 Center for Imaging Science, Johns Hopkins University; Institute for Computational Medicine, Johns Hopkins University, Baltimore, MD, United States of America; 7 Department of Biomedical Engineering, Johns Hopkins University, Baltimore, MD, United States of America; INSERM, FRANCE

## Abstract

We do not have a full understanding of the mechanisms underlying plasticity in the human brain. Mouse models have well controlled environments and genetics, and provide tools to help dissect the mechanisms underlying the observed responses to therapies devised for humans recovering from injury of ischemic nature or trauma. We aimed to detect plasticity following learning of a unilateral reaching movement, and relied on MRI performed with a rapid structural protocol suitable for *in vivo* brain imaging, and a longer diffusion tensor imaging (DTI) protocol executed *ex vivo*. *In vivo* MRI detected contralateral volume increases in trained animals (reachers), in circuits involved in motor control, sensory processing, and importantly, learning and memory. The temporal association area, parafascicular and mediodorsal thalamic nuclei were also enlarged. I*n vivo* MRI allowed us to detect longitudinal effects over the ~25 days training period. The interaction between time and group (trained versus not trained) supported a role for the contralateral, but also the ipsilateral hemisphere. While *ex vivo* imaging was affected by shrinkage due to the fixation, it allowed for superior resolution and improved contrast to noise ratios, especially for subcortical structures. We examined microstructural changes based on DTI, and identified increased fractional anisotropy and decreased apparent diffusion coefficient, predominantly in the cerebellum and its connections. Cortical thickness differences did not survive multiple corrections, but uncorrected statistics supported the contralateral effects seen with voxel based volumetric analysis, showing thickening in the somatosensory, motor and visual cortices. *In vivo* and *ex vivo* analyses identified plasticity in circuits relevant to selecting actions in a sensory-motor context, through exploitation of learned association and decision making. By mapping a connectivity atlas into our ex vivo template we revealed that changes due to skilled motor learning occurred in a network of 35 regions, including the primary and secondary motor (M1, M2) and sensory cortices (S1, S2), the caudate putamen (CPu), visual (V1) and temporal association cortex. The significant clusters intersected tractography based networks seeded in M1, M2, S1, V1 and CPu at levels > 80%. We found that 89% of the significant cluster belonged to a network seeded in the contralateral M1, and 85% to one seeded in the contralateral M2. Moreover, 40% of the M1 and S1 cluster by network intersections were in the top 80th percentile of the tract densities for their respective networks. Our investigation may be relevant to studies of rehabilitation and recovery, and points to widespread network changes that accompany motor learning that may have potential applications to designing recovery strategies following brain injury.

## Introduction

Exercise before injury or aging can induce beneficial plasticity, enhance brain resilience, and decrease its vulnerability to stress, depression [1], and degeneration [2]. With respect to injury (e.g. ischemia, or trauma) and rehabilitation, exercise can promote resilience to injury effects and promote recovery. Motor enrichment and exercise influence cognition [3, 4] and induce vascular and neuronal changes [5, 6]. The associated molecular and cellular changes could benefit the injured or aging brain through infusion of trophic factors such as brain-derived neurotrophic factor (BDNF), insulin-like growth factor-1 (IGF-1) or fibroblast growth factor-2 (FGF-2) [7, 8], neurogenesis [6], and enhanced myelination and regional brain volume in memory circuits [9].

Neuroanatomical and physiological studies support the dogma that the brain can be viewed as structurally and functionally dynamic, which is relevant in cases of damage, for example to the motor cortex [10]. While voluntary exercise, skill training, and forced limb use have been used extensively to this end, such strategies devised to work in humans need to be investigated carefully. Animal models can help reveal the mechanisms and interactions between different components of such interventions, e.g. the formation or enhancement of brain circuitry, or angiogenesis. Here we used high resolution magnetic resonance imaging as a translational tool to evaluate changes following a motor learning task. Our goal was to characterize the plasticity of brain circuits through behavioral, morphometric and microstructural properties (based on diffusion tensor imaging, or DTI).

Previous studies have evaluated brain plasticity in the mouse brain using cross sectional imaging following a motor task such as the rotarod [11], which tests motor coordination and balance; or more complex tasks involving learning and memory, such as the water maze [12]. In an example that comes close to our task Sampaio-Baptista and colleagues [13] examined the effect of a prehension task using rats. These studies have assessed either morphometry as a measure of gray matter plasticity [12], or fractional anisotropy (FA) as a measure of white matter plasticity [13], or a combination of morphometry and DTI for gray and white matter plasticity [14] [11]. There is a paucity of data using mouse neuroimaging, especially longitudinally and with multivariate approaches, to help understand the mechanisms behind the plasticity observed in human brains. Given the more robust ability to modify mouse behavioral abilities through gene editing or epigenetics [15–17], such studies would help advance our mechanistic understanding, and devise and test effective interventions.

We aimed to address this gap, along with the complementary values of two common scenarios in preclinical imagining, relying on rapid *in vivo* structural or prolonged *ex vivo* diffusion tensor based imaging protocols. We evaluated the plasticity following learning of a complex reaching movement, restricted to one limb, based on voxel based morphometry, cortical thickness and DTI microstructural changes. We cross examined our results against a mouse brain connectivity atlas [18] to identify more extended brain networks responsive to motor learning. Our investigation may be relevant to studies of rehabilitation and recovery, and have potential applications in designing and testing preventive strategies against degenerative conditions in the aging brain.

## Methods

### Animals

An animal cohort consisting of 24 C57BL/6 male mice, originating from The Jackson laboratory (Bar Harbor, ME), was divided into balanced groups between passive controls, and animals trained to perform a skilled prehension motor task. To keep the same experimental timeline animals were divided into three cohorts, balanced between controls and reachers (session 1: 3 controls, 3 reachers; session 2: 4 controls, 3 reachers; session 3: 5 controls, 6 reachers). The motor task has been previously described [19, 20] and restricts the animals to repeatedly using the right forelimb. Briefly, mice were food restricted to 85% of the baseline weight and trained in modified cages to reach for 45-mg dustless precision pellets (Bio-Serv). A training block consisted of 40 pellets at a distance of 1 cm, with each pellet presented one at a time. The animals underwent two blocks of 40 reaching attempts per training day, five days per week, and with one block per training day twice per week. Mice not undergoing training were treated just like the trained mice (allowed to run free in their cage, food restricted, fed the same pellets, and maintained on the same light/dark cycle). They were placed in the same training chambers for the same length of time as the mice undergoing motor learning and were given free access to the same food pellets placed on the floor of the cage as the mice undergoing training, but did not have to perform the complex reaching movement to access the food. Baseline imaging occurred when mice reached 85% of their weight prior to any exposure to prehension task. Mice were trained on the prehension task until plateau (23–27 days), reaching at least 50% success rate. Our behavioral success measure was defined as the percent of successful prehension attempts per pellet. Prehension was scored as a success when the mouse reached its forelimb through the slit, grabbed the pellet, and ate it without knocking it from its resting space, dropping it, or in any other way losing control of it. If not, the attempt was recorded as a miss. However, if the mouse did not touch the pellet, it was not counted as an attempt. The mice were monitored by manual observations. We have discarded the 25% most extreme performers based on behavior using the pure median average deviation estimator at day 23 (excluding values outside median ± 2* median average deviations), and balanced the groups analyzed at each time point to reduce potential bias, ending up with even groups of 9 animals.

The visreg package [21], http://pbreheny.github.io/visreg/) was used to graph the behavior data, including the 95% confidence intervals.

Final *in vivo* imaging occurred the day after the last training session. During imaging, mice were anesthetized with isoflurane (1%) in an oxygen–air mixture (1:3 ratio) via a vaporizer. All mice survived the two 40 min imaging sessions and recovered quickly after anesthesia.

At the end of the behavioral training period and immediately after the final *in vivo* MRI session, animals were sacrificed and brain specimens were prepared for *ex vivo* imaging following transcardiac perfusion fixation protocols similar to those described in [22] [23], tuned and evaluated by other groups as well [24] [25]. Mice were perfusion fixed with 4% PFA in phosphate-buffered saline (PBS), then the heads were removed and immersed in 4% PFA in PBS for 12 h at 4°C before being transferred to PBS. The specimens were kept in PBS (50 mL for each specimen; with weekly changes into fresh PBS), with 0.2 mM Gd-DTPA (Magnevist, Bayer HealthCare Pharmaceuticals Inc. Wayne, NJ, USA) for 2–3 weeks at 4°C. This procedure washed out residual fixative, which reduced tissue T2 and the signal-to-noise ratio of the acquired images, and introduced Gd-DTPA to enhance MR signals. Before *ex vivo* MRI, the specimens were placed into custom-built, MR-compatible tubes, filled with Fomblin (Fomblin Profludropolyether, Ausimont, Thorofare, NJ). This is an MR-invisible liquid for susceptibility matching and can also prevent dehydration. All animal procedures were performed with approval from the Johns Hopkins University Animal Care and Use Committee.

### Imaging

Animals were imaged *in vivo* at two time points separated by 25.4 ±1.2 days (23–27 days range), before and after learning to perform the skilled prehension task, i.e. reaching for food pellets. *In vivo* mouse brain MRI was performed on a 9.4 Tesla vertical bore NMR spectrometer (Bruker Biospin, Billerica, MA) equipped with a Micro 2.5 gradient system (100 G/cm maximum gradient strength), a manufacturer provided animal imaging probe, and a physiological monitoring system (electrocardiograph, respiration, and body temperature). A 20 mm diameter volume coil was used as the radiofrequency transmitter and receiver. Temperature was maintained by a heating block built into the gradient system. Respiration was monitored throughout the entire scan. Images were acquired using a three-dimensional (3D) T2-weighted fast spin echo sequence (FSE) with: echo time (TE)/repetition time (TR) = 40/700 ms, 16 mm x 16 mm x 16 mm field of view, and 128 x 128 x 80 matrix (resolution = 0.125 mm × 0.125 mm × 0.2 mm, echo train length = 4, number of average = 2, flip angle = 40°, bandwidth (BW) = 100 kHz). The total imaging time was ~40 minutes. T2-weighted FSE images were resampled to 125 μm isotropic resolution.

*Ex vivo* MRI-DTI of brain specimens was performed at approximately the same time after fixation (2 weeks), with max one-week difference, enforced by scanner availability and scan duration. We used the same 9.4 T NMR spectrometer and a 15 mm diameter volume coil as the radiofrequency transmitter and receiver. Images were acquired using a diffusion weighted 3D gradient and spin echo sequence [26] with: TE = 33 ms, TR = 900 ms, BW = 100 kHz, and 4 signal averages. The field of view and matrix size were 13.0 mm x 10.0 mm x 18.4 mm and 128 x 96 x 180, resulting in a native resolution of 0.1 mm x 0.1 mm x 0.1 mm. The spectral data were apodized by a symmetric trapezoidal function with 10% ramp widths on either side of the trapezoid and zero-filled before Fourier transformation. Six diffusion-weighted images (b value = 1700 s/mm^2^) and two non-diffusion-weighted images were acquired, and the total imaging time was 11 hours.

### Image analysis

The *ex vivo* 3D diffusion-weighted images were reconstructed on an off-line PC workstation using IDL (ITT Visual Information Solutions, Boulder, CO, USA). Average diffusion-weighted images (DWI) were generated from the six direction specific images acquired for each specimen. DTIStudio (www.mristudio.org) was used for log-linear tensor fitting. The tensor was then diagonalized to obtain three eigenvalues, based on which the FA [27] was calculated. The skull and other extraneous (non-brain) tissues were manually removed.

Voxel based morphometry relied on a high-performance compute cluster based pipeline [28]. This constructed minimum deformation templates based on *in vivo*, and *ex vivo* control animals separately, using a sequence of rigid, affine and symmetric diffeomorphic registrations implemented in ANTs [29, 30]. For the rigid and affine stages, DWI images were histogram-matched, and registered using a gradient step of 0.1 voxels, the Mattes similarity metric (32 bins, 10^−8^ convergence threshold, 20 iteration convergence window), in a multiresolution scheme with 2 down-sampling levels (6 and 4 times down-sampling), and smoothing sigmas of 4 and 2 voxels, and 3000 iterations.

We used a 0.5 voxel gradient step for the symmetric normalization (SyN); 3 and 0.5 regularization parameters for the velocity update and total warp field respectively, a cross correlation metric with a 4 voxel kernel radius, and full density sampling. We used a multi resolution scheme with 4 down-sampling levels (by factors of 8, 4, 2, and 1 –with 1 corresponding to the full resolution), and 4 smoothing sigmas (kernel standard deviations of 4, 2, 1, and 0 voxels), which the ANTs syntax represents as 8x4x2x1 for the spatial sampling, and 4x2x1x0 for the smoothing sigmas, respectively. We used maximum 4000 iterations per level.

Averaged DWI images were used to drive the registration for the *ex vivo* specimens. All images (*in vivo* and *ex vivo*) were registered to their respective minimum deformation templates using the same strategy to calculate deformation fields.

A mouse brain atlas [28] was used to label the minimum deformation template (MDT) with 332 labels, which were subsequently propagated onto the individual *ex vivo* images. Regional statistics were obtained for the volume, fractional anisotropy (FA), and apparent diffusion coefficient (ADC) parameters, which we have previously found to be sensitive markers for microstructural changes [31]. We computed regional volumes, and their corresponding microstructural properties, as well as cortical thickness in *ex vivo* images only, because of the clearer demarcation of cortical boundaries relative to the *in vivo* images, and because our atlas has been built using *ex vivo* DTI [32].

The segmented brain regions were used to estimate cortical thickness maps, relying on the displacement during diffeomorphic registration of the inner and outer cortical boundaries [33, 34]. For this purpose, we defined the outer shell as the sum of the isocortex regions, and the inner core as the sum of regions encapsulated by the corpus callosum.

Prior to statistical analyses images were smoothed with Gaussian kernels (250 μm standard deviation or sigma for *ex vivo* images, 313 μm for *in vivo*), aiming to respect the ratio of voxel sizes.

We determined morphometric and DTI changes based on fractional anisotropy and apparent diffusion coefficients using voxel based analysis (VBA), without the need for *a priori* defined regions. The *in vivo* and *ex vivo* images were analyzed separately, each in their own study specific template, because they present different resolution and contrast. Both the *in vivo* and *ex vivo* templates, as well as the statistical maps were however aligned into the same atlas space [32], to facilitate comparisons within groups, assuming that specimens for controls and trained animals shrink in a similar fashion.

The log Jacobian of the diffeomorphic deformation fields for both *in vivo* and *ex vivo* images was used to estimate local morphometric differences between animals trained to perform a skilled prehension task using the right paw, and age matched controls. FA and ADC maps were also compared voxel wise for the same groups to further characterize microstructural tissue changes.

### Statistical analyses

The Statistical Parametric Mapping (SPM) toolbox, version 12 [35, 36] was used to assess differences between trained and untrained animals, as well as the *in vivo* interaction term between time and training, implementing a repeated measures ANOVA design. To correct for multiple comparisons, we used a cluster-based false discovery rate (FDR = 0.05) and an initial cluster defining threshold of p = 0.05.

We hypothesized that changes in local volume and DTI parameters occurred along the primary motor pathways, as well as in circuits responsible for learning and memory. We compared statistical maps identifying plastic brain circuits against a reference connectivity atlas [18]. This atlas provided surrogates of connection probabilities, and each voxel in the atlas was assigned a value given by the total number of tracks originating from a seed region that pass through or terminate in that voxel. The comparison was done qualitatively using visualizations with multiple overlays for each VBA morphometric analysis (VBA-M), superimposed on tract density (TD) maps; and quantitatively—computing the overlap between significant clusters from *ex vivo* VBA morphometry and relevant regions from our anatomical atlas, as well as with the associated networks from the reference connectivity atlas. Specifically, we computed the percentage of the clusters found in each atlas region, and the percentage of atlas regions volume covered by the clusters. We estimated the percentage cluster in individual networks, with respect to the size of the cluster, and to the size of the network. We estimated the median and maximum tract densities for the intersections between clusters and atlas regions. Finally, we computed the number of significant voxels in the upper 80th percentile for relevant TD maps.

### Visualization

FSLeyes (https://fsl.fmrib.ox.ac.uk/fsl/fslwiki/FSLeyes) was used for cross sectional visualization of statistical parametric maps overlaid on the study specific templates based on FSE for *in vivo* and on FA for *ex vivo* images. The coronal slice visualizations in **Figs 3–5** show the anatomical/or FA image as the bottom layer, uncorrected statistics as the next (shown in yellow), while corrected statistics are shown overlaid, as the top layers, using distinct color schemes (blue for *in vivo* VBA-M at the end of learning; green for the *in vivo* VBA-M interaction term; orange for the *ex vivo* VBA-M). **Fig 7** uses the yellow color scheme for showing tract density, and the corrected statistical maps reuse the same color coding as in **Figs 3–5**. Avizo (ThermoScientific, Houston USA) was used for volume rendering.

## Results

### Behavioral assessment

We evaluated the impact of learning a complex motor task on the brain morphometry and microstructure, using mice trained to reach for food pellets using the right paw, and imaged *in vivo* with structural MRI and *ex vivo* with DTI. Our results confirm that plasticity manifests at the level of behavior, brain morphometry, as well as microstructural properties.

**Fig 1:**
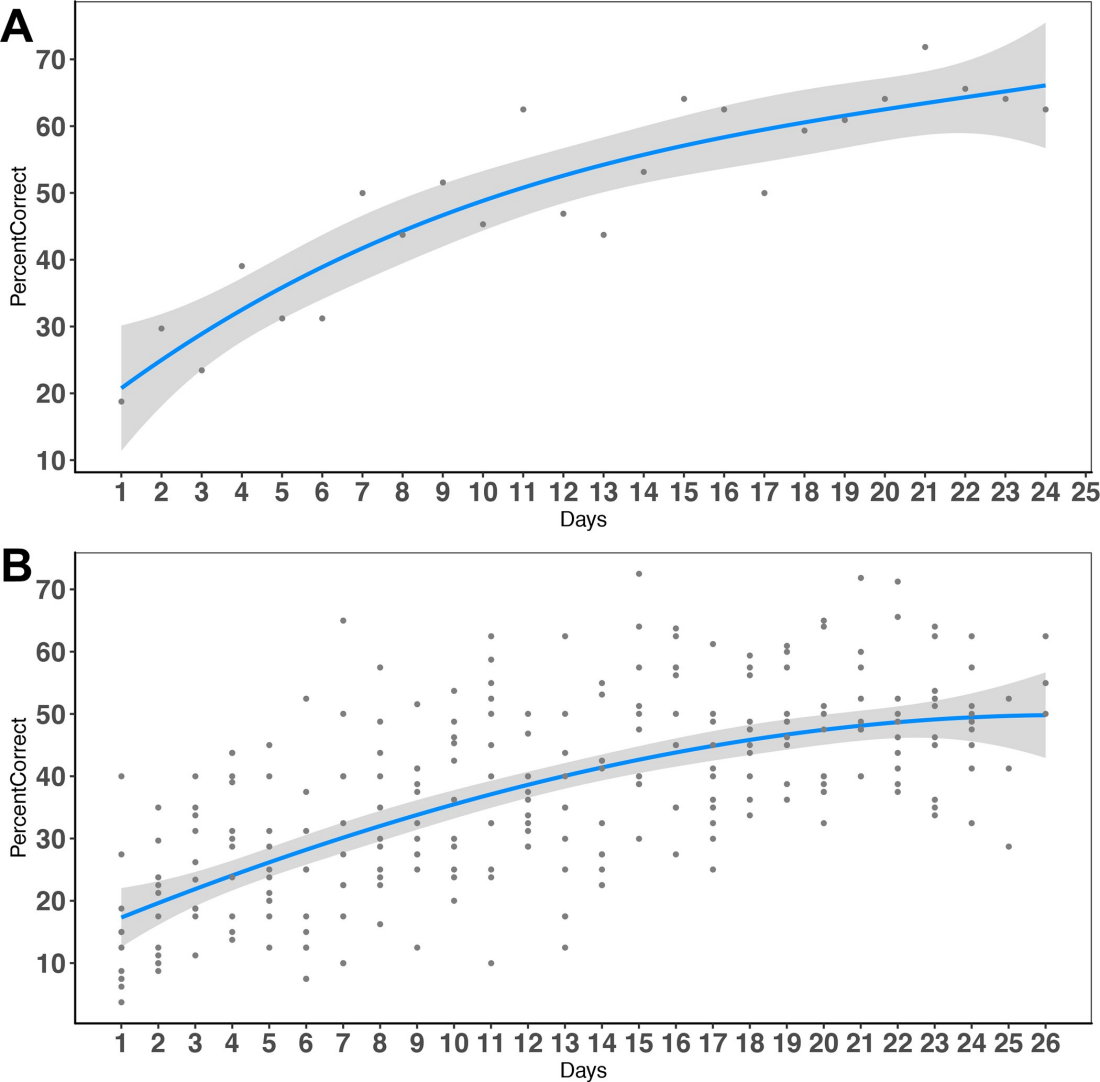
Skilled prehension task performance. A. Learning performance in a representative mouse. B. Average learning performance for all mice trained in the skilled prehension task, modeled with a 3^rd^ order polynomial. The average on the last day of training was 55.13±8.24%.

The percentage of successful attempts in the skilled prehension task improved over the ~25 days of training (range 23–27 days; mean 25.4±1.2), from 14.77±10.75% on the first day to ~55.13±8.24% correct on the last day of training (**Fig 1**).

### Population averages

**Fig 2:**
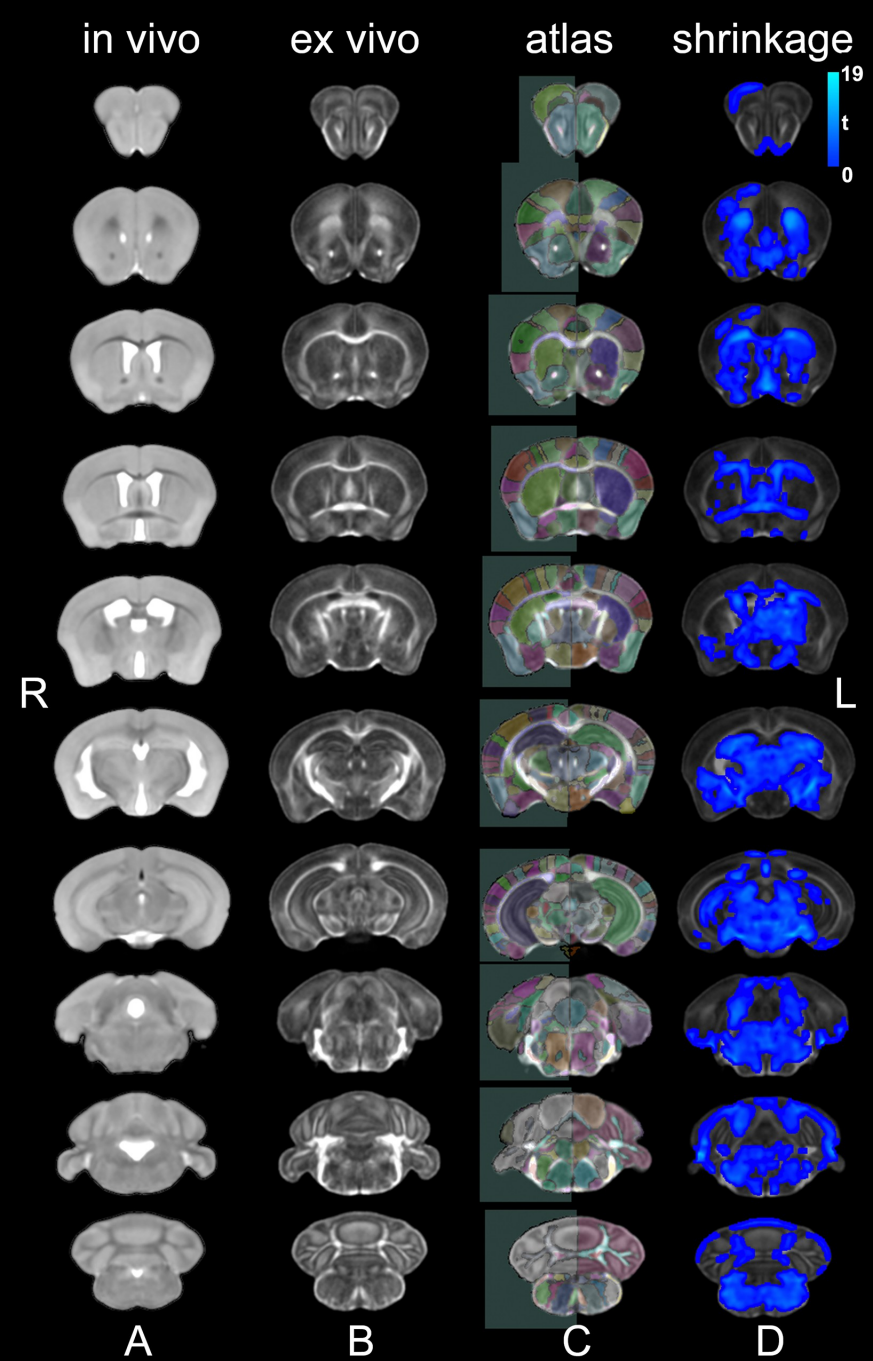
*In vivo* and *ex vivo* study specific templates normalized into a 3^rd^ generation Waxholm space. We created study specific templates for *in vivo* T2-weighted FSE images (A) and *ex vivo* DTI parametric images. The transforms used to register the DWI contrasts were re-used to generate the FA template, with good contrast for gray/white matter boundaries (B). We automatically segmented 332 regions for the *ex vivo* specimens based on a reference atlas [28] (C). A comparison of the *in vivo* versus *ex vivo* images revealed areas of shrinkage (blue) in control specimens. The statistical *t*maps were cluster-corrected for multiple comparisons, using an FDR threshold of 5%, and an initial cluster forming threshold significance level of 0.05 (D).

We produced *in vivo* and *ex vivo* population averages for the control animals, to serve as study specific reference spaces for analyzing differences due to learning, and to address biases due to shrinkage (t = 4.04 ± 1.92; mean ± standard deviation) in the fixed specimens (**Fig 2**).

### *In vivo* voxel based morphometry

**Fig 3:**
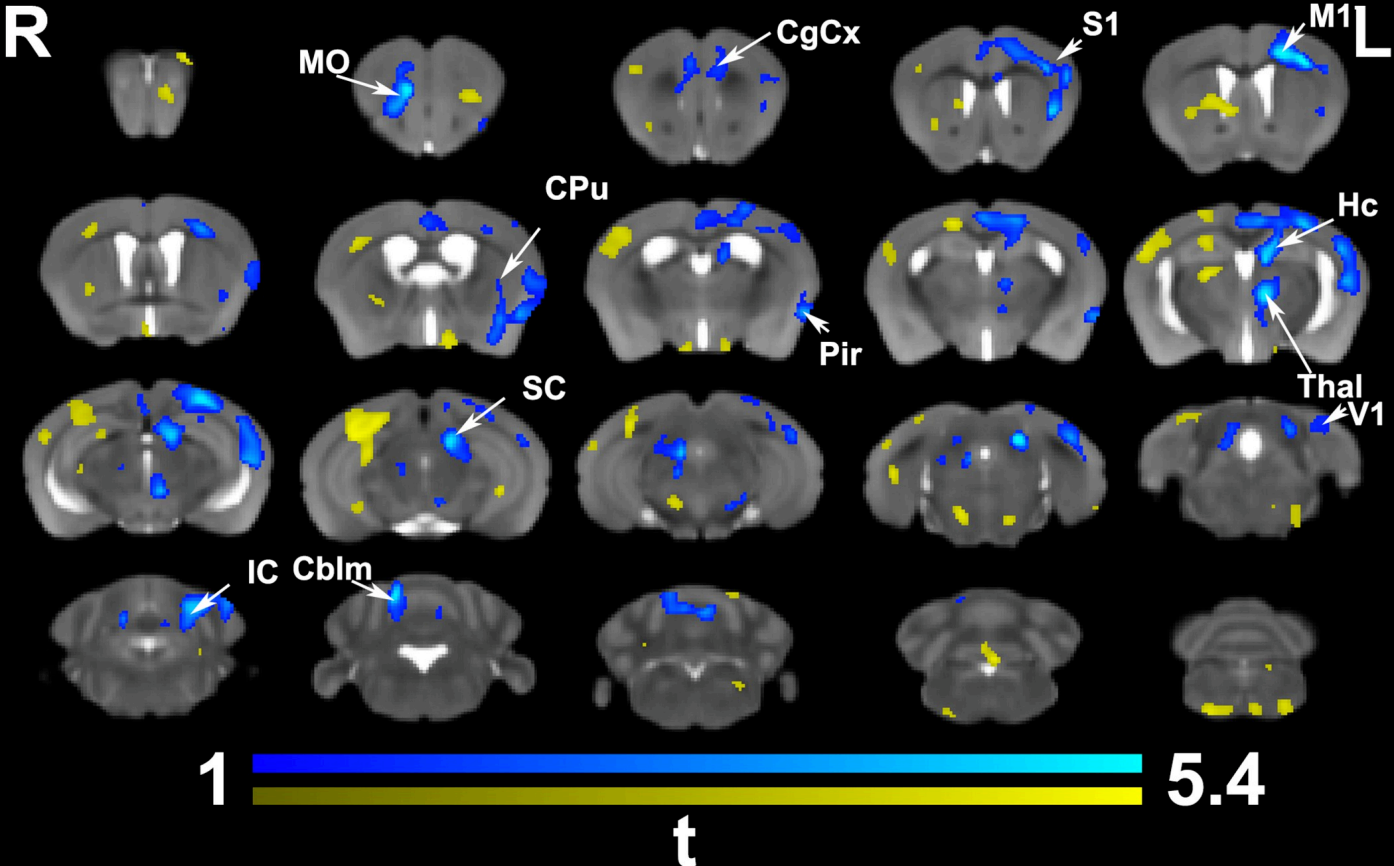
*In vivo* voxel based morphometry identified areas of significant enlargement in trained animals relative to the controls. These areas were found in the bilateral medial orbital cortex (MO) and cingulate cortex (CgCx) (A32), as well is in the contralateral (left) cingulate cortex (A24, and 29), primary motor (M1), somatosensory (S1), caudate putamen (CPu), and hippocampus (Hc); and the visual (V1) cortex. In addition, clusters of hypertrophy covered areas of the thalamus (Thal; e.g. the parafascicular and mediodorsal nuclei), the superior (SC) and inferior colliculi (IC), and cerebellum (Cblm). The ipsilateral piriform cortex (Pir) was enlarged. Results are presented as *t*maps, FDR cluster-corrected for multiple comparisons using an initial cluster forming threshold of 0.05 significance and the whole brain as a mask (blue color). Uncorrected statistical maps (shown in yellow) suggested involvement of the ipsilateral hemisphere as well.

Morphometric differences based on *in vivo* imaging at the end of the learning process (**Fig 3**) were significant after cluster based correction, predominantly in regions located in the contralateral hemisphere. The contralateral primary and secondary motor (M1, M2) and somatosensory (S1, S2) cortices, the caudate putamen (CPu) and hippocampus (Hc) were enlarged, as well as the temporal association, and visual cortex areas. In addition, thalamic nuclei also appeared enlarged unilaterally, in particular the parafascicular, centromedial, and mediodorsal nuclei. The midbrain precuneiform nucleus was enlarged. Bilateral changes were observed in the orbital cortex (lateral, and ventral), the piriform cortex, and the cingulate (areas 24, and 32), and in the cerebellum. The i*n vivo* analysis at the end of the learning process identified two significant clusters with a total coverage of 12067 voxels, out of the average study template brain volume of 220204 voxels, or 5.5% of the brain volume (3.7% in the 1^st^, and 1.8% in the 2^nd^ cluster). The uncorrected statistics suggested involvement of the ipsilateral motor and somatosensory areas as well.

**Fig 4:**
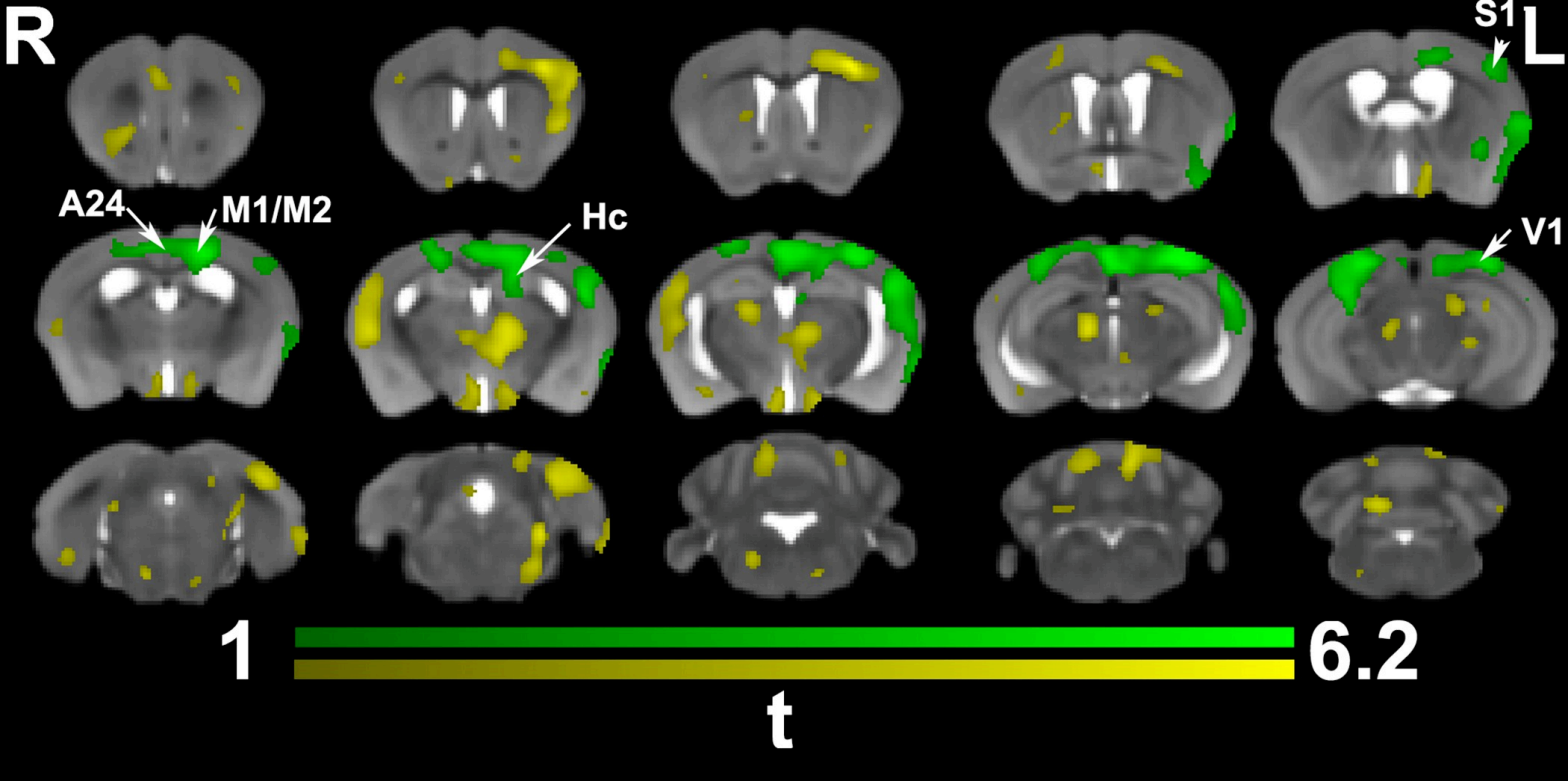
*In vivo* voxel based morphometry identified a significant interaction between time and training. Trained animals showed significant enlargement prominently in the contralateral (but also ipsilateral) cingulate cortex (A24, and 29), M1, S1, and the visual cortex (V1). The contralateral hippocampus, and corpus callosum, under the M1 were also enlarged. Results are presented as *t*maps, FDR cluster-corrected for multiple comparisons using an initial cluster forming threshold of 0.05 significance, and the whole brain as a mask (green color). Uncorrected statistical maps (yellow) suggested involvement of the ipsilateral hemisphere.

When examining the interaction between time and group (trained versus non-trained) (**Fig 4**) significant morphometric changes were found largely in the same cortical areas seen when comparing trained animals and controls, but missed the more rostral areas of the orbital cortex, as well as the thalamic, midbrain and cerebellar components seen at the end of training. These results indicated a role for both the ipsilateral and contralateral M1, M2, S1 (forelimb and hindlimb) cortices, the cingulate cortex (A24), as well as the hippocampus (Hc). The interaction term identified one cluster covering an area of 9142 voxels, or 4.2% of the brain volume, overlapping the results at the end of training by 36% (Dice coefficient). The uncorrected statistics covered a larger area (13480 voxels, or 6.6% of the brain volume), suggesting a role for the more rostral motor areas, the visual cortex, the parafascicular, centromedial, and mediodorsal thalamic nuclei, the hypothalamus, and the cerebellum.

### *Ex vivo* regional and voxel based morphometry following learning

The total brain volume only showed a trend (p = 0.1) towards being enlarged in reachers (450± 11 mm^3^) relative to controls (441±12 mm^3^). Regional differences based on morphometric and DTI estimates did not survive FDR correction for the whole set of 332 regions, however the uncorrected statistics suggested enlargement of the left parietal cortex posterior area (13%, p = 0.006, t = 3.2, df = 16; 12.3±0.8 10^−3^% brain volume in reachers, versus 11.0± 1.1 10^−3^ in controls), and 3% in primary somatosensory cortex (upper lip region, S1UL) (p = 0.02, t = 2.6, 0.57±0.02% of brain volume in reachers versus 0.55±0.01 in controls).

**Fig 5:**
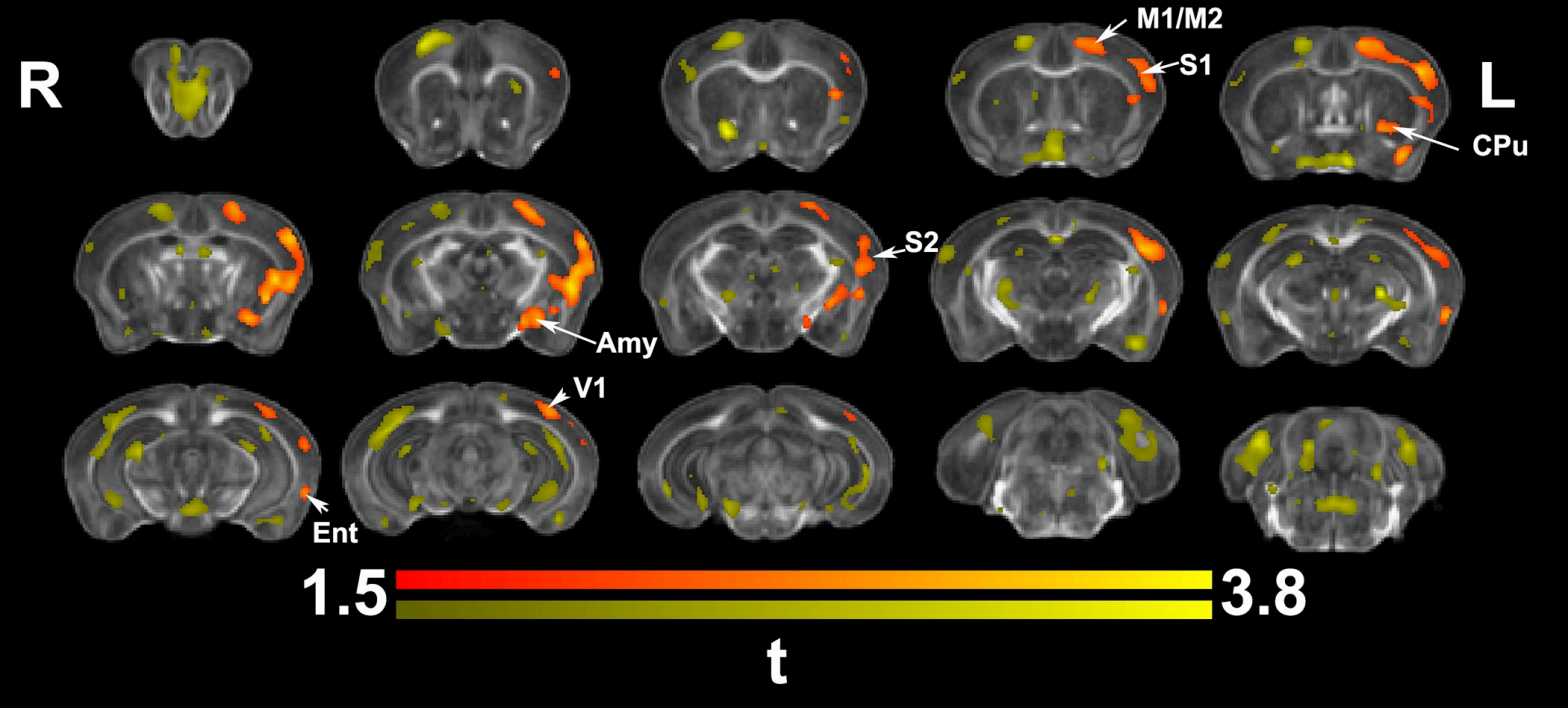
*Ex vivo* voxel based morphometry identified areas of significant enlargement in trained animals relative to controls. These were located in the contralateral (left) M1, S1, S2, CPu, amygdala (Amy), as well as the visual (V1) and entorhinal cortex (Ent). Results are presented as *t*maps, FDR cluster-corrected for multiple comparisons using an initial cluster forming threshold of 0.05 significance and the whole brain as a mask (orange color). Uncorrected statistical maps (yellow), suggested involvement of the ipsilateral hemisphere as well, and a role for the hippocampus.

*Ex vivo* analysis of morphometric changes in reachers (**Fig 5**) also revealed hypertrophy in the contralateral (left) M1, M2, S1, S2, CPu, primary visual cortex, as well as novel clusters in the entorhinal cortex, amygdala, and the auditory cortex (compared to *in vivo* findings). White matter tracts involved included the corpus callosum and optic tracts. *Ex vivo* based morphometry identified one cluster covering an area of 7604 voxels, or 1.9% of the brain volume of 410804 voxels, overlapping the *in vivo* results at the end of training by 6% (Dice coefficient).

Together, our voxel based analyses identified volume changes after learning a skilled prehension task in circuits involved with sensory motor processing, and integrating information from a limbic component.

We next compared cortical thickness estimates based on *ex vivo* imaging, since this provided a better definition of the anatomical regions, particularly for corpus callosum/ isocortex boundaries. These results did not survive multiple comparison correction, but the uncorrected statistics suggested thickening of the contralateral somatosensory (especially forelimb and jaw regions) and visual cortices (V1, V2), as well as for the bilateral olfactory/pirifom cortex, supporting the results of voxel based morphometry (**S1 Fig**).

### *Ex vivo* regional and voxel based dti analyses following learning

Regional analyses revealed that the FA for the cerebellar white matter was higher in reachers relative to control animals (5%, p = 0.02, t = 2.5, df = 16; 0.53±0.02 in reachers, versus 0.51±0.02 in controls). Conversely, the ADC was lower in reachers for the superior cerebellar peduncle (8%, p = 0.005, t = -3.1, df = 16; 0.35±0.02 10^−3^ mm^2^/s in reachers, versus 0.38±0.02 10^−3^ mm^2^/s in controls), and the fastigial nucleus (10%, p = 0.003, t = -3.6, df = 16; 0.36±0.03 10^−3^ mm^2^/s in reachers, versus 0.40±0.02 10^−3^ mm^2^/s in controls).

**Fig 6:**
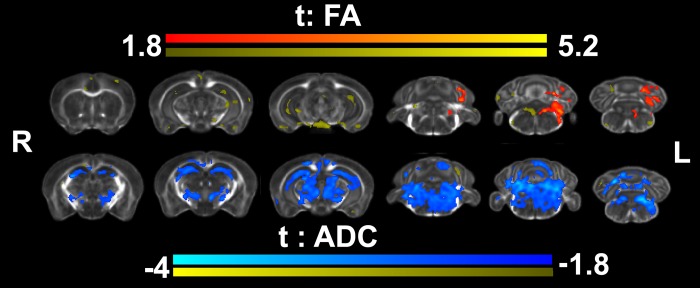
Voxel based analyses for fractional anisotropy (FA) and apparent diffusion coefficient (ADC) detected increased FA and decreased ADC in trained reachers relative to controls. These occurred mostly in caudal regions such as the cerebellum and brainstem (ADC), but largely missed the motor and sensory cortices, although decreased ADC values were detected in the corpus callosum, below M1. Results are presented as *t*maps, FDR cluster-corrected for multiple comparisons using an initial cluster forming threshold of 0.05 significance, and the whole brain as a mask. Uncorrected statistical maps are also shown in yellow, suggesting microstructural changes in the hippocampus and isocortex.

We next used voxel based analysis to evaluate microstructural changes in trained animals based on FA (VBA-FA) and ADC (VBA-ADC) (**Fig 6**). VBA-FA revealed FA increases localized to the caudal aspects of the brain, the cerebellum, and inferior cerebellar peduncle. Relaxing the statistical threshold (to p<0.05) suggested changes also in the hippocampus, S1, V1, cingulate cortices. VBA-ADC revealed significant and more extensive ADC changes, in areas of the hippocampus and thalamus, deep mesencephalic nuclei, the red nucleus, cerebellum and its connections, as well as the pons reticular formation. White matter tracts showing reduced ADC included the corpus callosum, internal capsule, medial lemniscus, the cerebellar white matter, and cerebellar peduncles. Together, these findings suggest microstructural changes occurred in response to motor training in both gray and white matter regions.

**Fig 7:**
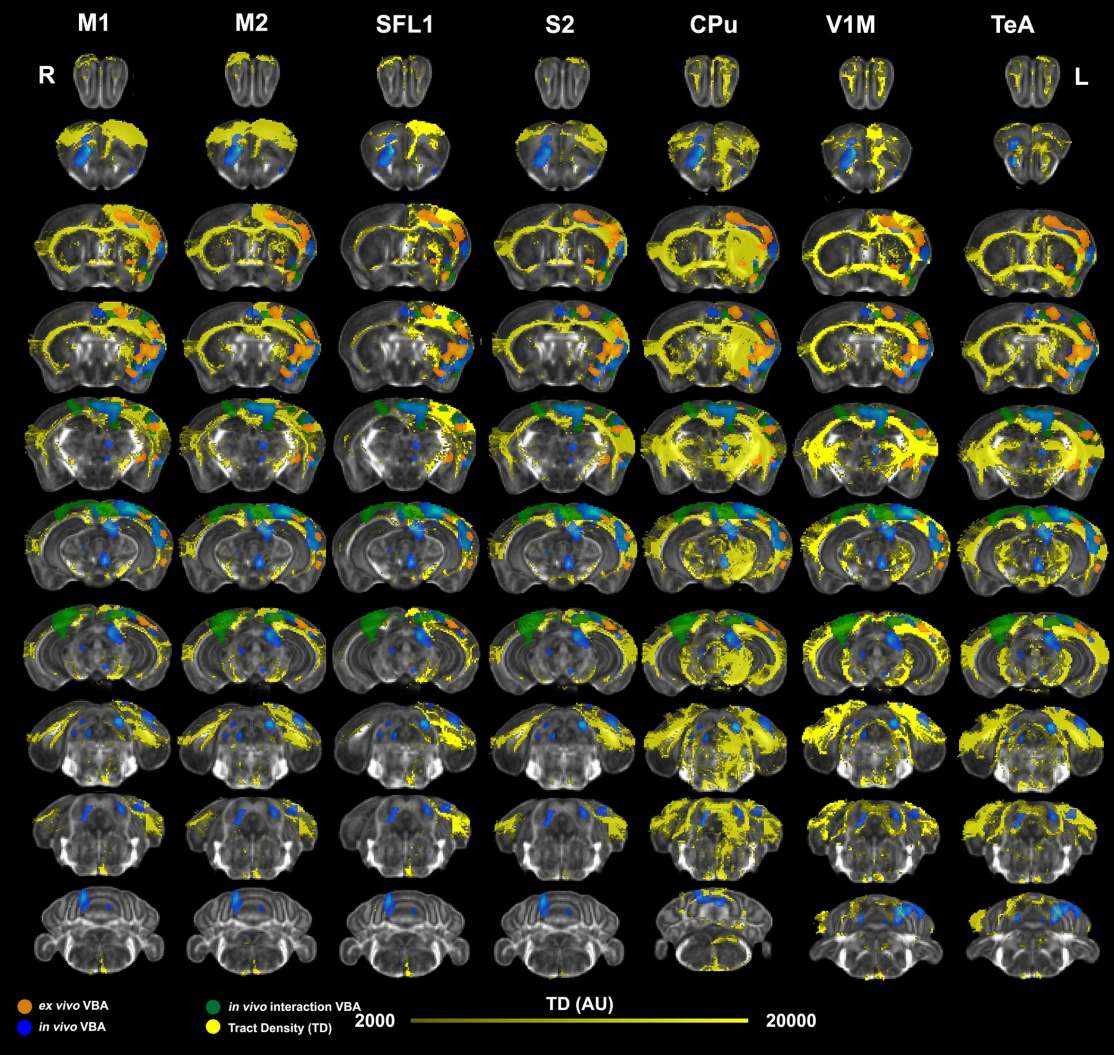
Mapping of *ex vivo* VBA-M and tract based connectivity maps. To verify the overlap between morphometric changes detected *ex vivo* and circuits relevant to motor learning, we registered tract based connectivity maps [18] for individual seed regions to the minimum deformation template from our *ex vivo* study. We examined networks seeded in regions found to be enlarged in the contralateral (left) hemisphere, including the primary and secondary motor cortices (M1, and M2), as well as the primary and secondary somatosensory cortices (S1 –the forelimb region, S2), caudate putamen (CPu), V1M primary visual cortex (monocular); and the temporal association cortex (TeA). Significant clusters for *in vivo* morphometry at the terminal point are shown in blue, the interaction of time by group (trained versus not trained) in green, and the morphometric results from *ex vivo* specimens are shown in orange. These clusters showed good overlap with the tract density maps (TD).

We compared voxel based analysis results with tract density maps reconstructed based on a connectivity reference atlas (**Table 1**, and **Fig 7**) [18], registered to our study specific templates. We reconstructed tract density images for all atlas regions, but only show those tracts intersecting significant clusters identified by *ex vivo* voxel based morphometry (which were the most conservative in terms of the extent of detected changes).

**Table 1:** Comparison of *ex vivo* VBA-M and tract based connectivity maps based on a reference atlas [18] providing anatomical regions and associated tract densities (TD) for 332 ROIs.

Abbrev.	Cluster ∩ ROI	Cluster ∩ ROI (% ROI)	Max TD (AU)	Median TD (AU)	Cluster ∩ Network (% Cluster)	Cluster ∩ Network (% Network)	>80 percentile of TD
	(% Cluster)						
**M1**	**6.85**	**13.23**	1.27E+08	7300.78	**89.11**	2.82	**37.23**
**M2**	0.59	1.07	7.81E+07	3855.64	**84.69**	2.65	26.2
**S1**	0.53	**9.28**	1.29E+08	10904.67	**83.65**	2.71	**32.75**
**CPu**	**14.17**	**10.06**	8.97E+07	3195.99	82.18	1.58	25.89
**S1BF**	**9.23**	**15.46**	6.05E+07	9155.23	**79.3**	2.94	**40.48**
**V1M**	0.08	0.28	9.97E+07	6929.78	77.67	2.51	26.56
**S1FL**	3.91	**15.82**	1.42E+08	2899.99	77.52	3.6	26.18
**AuD**	1.71	**16.46**	5.01E+07	8671.08	77.5	2.72	26.21
**V1**	0.14	3.79	3.16E+07	4064.39	**77.46**	2.4	21.61
**S2**	**11.22**	**31.16**	1.24E+08	18128.94	75.49	2.87	**35.05**
**S1HL**	**5.96**	31.28	8.22E+07	2775.28	75.24	4.3	29.12
**V2L**	2.46	5.32	4.40E+07	4589.09	**74.78**	2.47	21.44
**S1UL**	**11.81**	**35.96**	1.54E+08	8640.17	73.4	3.43	32.73
**Pir**	**6.09**	1.73	1.59E+07	412.1	**73.01**	1.6	20.92
**TeA**	0.03	0.14	4.68E+07	3821.42	**70.91**	2.07	20.27
**Ins**	3.68	3.24	2.54E+07	1399.28	69.77	2.3	19.61
**Ect**	0.04	0.13	6.59E+07	3809.28	68.91	2.08	20.8
**AuV**	1.16	5.18	4.44E+07	5253.91	68.78	2.68	23.11
**V1B**	2.32	**16.43**	2.26E+07	3044.9	68.46	2.59	19.4
**PRh**	0.46	3.76	7.05E+07	3521.97	66	2.19	19.29
**Au1**	1.85	3.27	4.12E+07	3772.89	63.81	2.3	17.02
**S1Tr**	0.13	3.22	3.02E+07	4649.03	61.35	5.36	17.64
**GP**	0.38	0.74	3.74E+07	3484.7	59.11	1.57	14.3
**S1DZ**	0.42	4.33	3.66E+08	6821.74	56.64	5.04	11.93
**Amy**	7.35	8.04	3.36E+07	511.08	54.01	1.31	15.07
**VPal**	0.96	0.15	8.61E+07	3464.94	53.24	1.34	15.02
**ot**	0.75	0.29	2.71E+08	6826.12	52.93	1.55	11.76
**S1J**	0.91	4.25	9.03E+08	6278.1	51.08	4.28	12.68
**VCl**	1.46	0.84	1.96E+08	10967.93	47.5	2.25	10.51
**DLEnt**	1.42	2.29	7.10E+07	2564.6	46.05	2.16	12.32
**Preopt**	0.03	0.07	3.19E+07	990.39	43.29	1.05	11.1
**Hyp**	0.14	0.24	1.86E+07	762.85	41.6	1.01	7.56
**DCl**	0.53	3.38	4.58E+08	19983.45	40.93	2.57	9.53
**S1Sh**	0.01	1.23	6.84E+07	4307.68	40.01	5.1	7.13
**cc**	1.21	0.55	7.07E+07	2143.26	37.09	1.13	7.55

The left most column shows the atlas regions (abbreviations) intersected by significant clusters identified by *ex vivo* VBA-M. The percentage of the total cluster found in each atlas region was highest (14%) for the CPu, followed by S1/S2 (~ 10%), and M1 (7%). The percentage of atlas regions covered by the VBA-M clusters was >30% for S1/S2, and >10% for M1 and CPu. We estimated the median and maximum tract density for the overlap between clusters and atlas regions, and the percentage cluster in individual networks, with respect to the size of the cluster, and the size of the network. More than 80% of the clusters were found to overlap with networks for M1, M2, S1, although these clusters occupied a small percentage of the extensive connectivity maps for these regions. The number of significant voxels in the upper 80th percentile for the individual tract density maps was ~40% for M1/S1. Abbreviations: M1: primary motor cortex, M2: secondary motor cortex, S1 primary somatosensory cortex, CPu: caudate putamen, BF: barrel field, V1M: primary visual cortex (monocular), FL: forelimb; AuD: auditory cortex; S2: secondary somatosensory cortex; HL: hind limb; UL: upper lip; V2: secondary visual cortex; Pir: piriform; TeA: temporal association area; Ins: insula; Ect: ectorhinal cortex; PRh: perirhinal cortex; Tr: trunk; GP: globus pallidus; DZ: dysgranular; Amy: amygdala; J: jaw; Cl: ventral claustrum; DLEnt: dorsolateral entorhinal cortex; Preot: preoptic; Hyp: hypothalamus; DCl: dorsal claustrum; Sh: shoulder; cc: corpus callosum.

The tract density (TD) maps (**Fig 7**) were seeded in the contralateral primary and secondary motor cortices (M1, M2), the primary somatosensory cortex (S1, its forelimb region), the secondary somatosensory cortex (S2), caudate putamen (CPu), primary visual cortex (monocular vision, V1M), and the temporal association cortex (TeA). A qualitative evaluation showed good overlap between morphometric changes in circuits connecting the contralateral S1, S2, M1, M2 and V1 areas. Interestingly, we found that most of these networks also project to ipsilateral areas, supporting the validity of ipsilateral findings from VBA-M.

We also evaluated quantitatively the overlap between brain networks shown to have plasticity following learning based on significant *ex vivo* VBA-M clusters, and atlas based tract density maps. We identified based on this atlas a set of 35 network nodes which showed plasticity following motor learning. This included prominently the primary and secondary motor and sensory cortices, as well as the caudate putamen, visual cortex, and the temporal association cortex. Our quantitative analysis showed that 89% of the significant clusters’ extent identified by VBA-M belonged to a network identified based on seeding tracts in the contralateral M1, and 85% were also part of a network identified based on seeding in the contralateral M2. The caudate putamen (CPu) and primary somatosensory (S1) networks were also represented at the 80% level. Approximately 40% of these cluster by tract intersections for M1, S1BF (barrel field) and S2 were in the 80th percentiles of the network densities. These top ranked networks were followed by those for the M2, S1, CPu, primary visual (V1M) and auditory cortex (30% of voxel in 80th percentile), and ~20% of ectorhinal cortex and insula, temporal association cortex, while V1, the entorhinal cortex and claustrum were represented at the 10% level.

Together these analyses suggested a good overlap between VBA-M results and networks identified as important based on the reference connectivity atlas. Our results confirmed that morphometric changes occurred in areas connecting not just M1, M2, and S1, S2, but involved more extensive thalamocortical-circuits and networks with roles in associative functions. These results indicate plasticity in circuits involved with sensory-motor function, and with associative learning and decision making following a skilled prehension task acquisition.

## Discussion

### Motivation

Animal models can help dissect the mechanisms behind brain plasticity following injury and repair, and help devise and test strategies for building a reserve conferring enhanced brain resilience to injury and degenerative processes. Importantly, animal studies can be conducted in well controlled environments, with subjects having the same genotypes and experiences (e.g. environmental enrichment or exercise routine). Such studies can help us understand how genes modulate brain plasticity, or the selective vulnerability of brain networks, and highlight important network nodes and pathways that can be targeted for interventions. For example, Sampaio-Baptista and colleagues [13] used 72 rats in a motor learning skill and found microstructural changes using *ex vivo* imaging. This study found increased FA in the corpus callosum overlying the motor cortex, cingulum and external capsule; but did not include a morphometric analysis.

The present study examined the extent to which we can detect brain plasticity following a complex reaching task restricted to the right forelimb. We quantified how it manifests at the level of behavioral, morphometric, as well as microstructural changes revealed through DTI protocols.

### *In vivo* and *ex vivo* voxel based morphometry

Our *in vivo* and *ex vivo* voxel based analyses corroborated to support enlarged local volumes in the contralateral hemisphere, primarily in the somatosensory and motor cortices (M1, M2, S1, and S2), and also in the caudate putamen and the visual cortex. Interestingly, hippocampal volume changes were significant in the *in vivo* data, while the *ex vivo* data showed changes only in the uncorrected statistics.

Both *in vivo* and *ex vivo* morphometric analyses revealed expected contralateral increases in local volumes for the motor and sensory cortices. We also found ipsilateral changes, which have been more controversial, but have been reported in humans based on fMRI activations during the execution of a unilateral task, where effects have been found to depend on the precision of the required task [37]. The changes in volume may be attributed to existing interhemispheric connectivity, which is enhanced through the learning process, and possibly to rewiring. Moreover, enhanced ipsilateral inhibitory activity can also lead to increased volume.

Besides these expected changes in the primary and secondary motor and somatosensory cortices at the end of training, more extended networks involved the cerebellum, motor related areas of the midbrain (seen *in vivo* only), as well as the cingulate cortex, the hippocampus, amygdala, temporal association areas, and thalamic nuclei. These included the parafascicular, mediodorsal, and centromedial nuclei. The parafascicular receives input from layer 5 of the limbic, association, and sensory-motor cortices, providing major excitatory input to the caudate putamen, while also sending projections to the cortex. The parafascicular nuclei are thus thought to form networks that shape the caudate putamen output, to mediate the correct action selection in a sensory motor context [38]. The mediodorsal nucleus, and lateral habenula have been associated with adaptive behaviors [39, 40]. The centromedial nucleus projects to limbic and sensory motor regions in the rostral forebrain, which suggests a role in integrating affective, cognitive and sensory motor information to improve goal directed actions [41].

Compared to findings at the end of training, the *in vivo* interaction of time by group (trained versus non trained) indicated more bilaterality, suggesting that regional volumes prior to training may predict the ability to respond to interventions.

Thus, voxel based morphometric analyses identified plasticity in circuits responsible for sensory motor function, as it is integrated with learning and memory during training. Our data support that changes occurred in circuits involved in both declarative and procedural memory circuits.

### *Ex vivo* voxel based dti analyses

Previous studies [13] have suggested that learning a motor skill induces microstructural white matter changes in FA, which may be due to learning-related increases in myelination. In our study, we found predominant changes in caudal regions such as the cerebellum and pons (reticular formation) survived FDR corrections in both VBA-FA and VBA-ADC analyses. Only the *ex vivo* ADC analyses detected hippocampal changes at a microstructural level. Uncorrected statistics suggest that future studies may detect FA changes in the hippocampus, and relevant areas of the isocortex as well. These data support the VBA-M results, suggestive of changes in circuits integrating learning in a sensory motor context.

### Volumetry to connectome mapping

Together, the *in vivo* and *ex vivo* results suggested plasticity in circuits relevant to exploitation of learned association and decision making, to select and shape actions in a sensory-motor context. To confirm that observed changes occurred in circuits related to motor learning and memory formation, we mapped a reference connectome atlas into our *ex vivo* study specific template [18]. By registering this tractography based connectivity atlas to our data sets, we verified that plasticity due to learning a skilled prehension task occurred in a set of 35 regions including the primary motor cortex (M1, 7% of the cluster), the caudate putamen (CPu, 14% of the cluster), the somatosensory cortices (S1BF, 9%; S2, 11% of the cluster), as well as the visual (V1) and temporal association cortex (TeA). The significant clusters intersected networks seeded in M1, M2, S1, and CPu at levels of ~80% or higher. 89% of the significant cluster belonged to a network seeded in the contralateral M1, and 85% to one seeded in the contralateral M2. V1 was represented at 77% level. 40% of the M1 and S1BF cluster by network intersections were in the top 80th percentile of the tract densities for their respective networks. VBA-M provided significant clusters which were part of sensory motor networks, as well as a limbic component modulating learning and memory, leading to improved action control in a sensory motor context.

### Considerations on experimental design

We have used protocols for preparing brain specimens similar to [22] [23], tuned and evaluated by other groups as well [24] [25]. We have imaged specimens in the skull to avoid shape distortions and tissue damage [42]. We computed T1 and T2 relaxation maps after soaking specimens in PBS and 0.5 mM Gd-DTPA between fixation and scan time between 7–14 days reduced T1 to ~100 ms, and T2 to ~25 ms. Future studies may consider further optimizing the times for fixation and soaking in PBS+GdDTPA to increase contrast [43] and ensure stability of the tissue relaxation parameters. For example, [44] found that 24 hours fixation, 24 hours soaking and 5 days storage were required for achieving a stable signal to noise ratio (SNR). On the other hand, [45] showed that the brain undergoes morphometric changes, primarily atrophy with prolonged fixation and storage times, at a rate of 3% per month, and importantly, that these changes occur nonuniformly throughout the brain. The nonuniformity may be explained by region dependent cellular composition, or the proximity to skull and fluid filled areas. Thus, [45] recommended imaging between 7 and 40 days to keep differences within 1%, or between 1–6 months for keeping volume differences within 5%; or alternatively to image after storing between 7–14 days or between 1–3 months PBS. In our own study, we found changes of 7% in the caudate putamen volume, and 5% in the hippocampus between *in vivo* and *ex vivo* estimates, which may be overestimated due to differences in resolution between *in vivo* and *ex vivo* scans. Independent studies on preparing specimens for imaging and pathology [46, 47] recommended neutral buffered formalin fixation over paraformaldehyde-lysine-periodate and paraformaldehyde fixation, and acknowledged shrinkage issues. While zinc based fixation methods may confer less shrinkage, they also resulted in a higher degree of deformation [47]. Importantly, maintaining consistency in the fixation protocol, and the interval between fixation and imaging will help reduce experimental biases. To help control for variability due to time dependent changes in specimen properties, we have used such a systematic approach, treating all specimens the same way in terms of fixation and storage time (12 hours, and ~14 days storage).

Our imaging protocol used 4 signal averages due to the use of phase cycling to remove stimulated echoes from imperfect 180^o^ radio frequency pulses in our sequence, which included multiple refocusing pulses. We later developed an optimized gradient spoiler scheme that enabled us to use only 2 signal averages. Future protocols should use more diffusion directions, and higher spatial resolution to enhance sensitivity to white matter changes as detected previously in rats [13], to increase our ability to examine mouse brain tractography and connectivity. Accelerated imaging protocols are needed the achieve the high angular and spatial resolution required to detect plasticity in mice, and our efforts are moving towards using compressed sensing to provide 8x acceleration [48].

### Limitations

A limitation of our study is that we cannot discern whether the changes we observed were due to learning, limb activity, or enrichment effects due to task exposure. Future studies focused on correlative analyses of imaging biomarkers with behavioral traits could help establish the mechanistic relationships between structural changes and behavioral performance. A second limitation is that we have compared results based on two very different imagining protocols, thus some of these differences are methodological and some are biological. Nevertheless, our study used two common approaches in preclinical imaging, and illustrated some of their relative strengths and weaknesses in the context of imaging plasticity. The third limitation is that only males were included in this study. It would be interesting to evaluate whether a possibly enhanced interhemispheric connectivity results in different responses in females.

Our study adds knowledge on morphometric and white matter microstructural plasticity [13, 14, 49], and was powered to detect a Cohen effect size of 1.23, for 9 animals/group, at a significance level of 0.05 with 80% power. We measured effect sizes on the order of 1.50 for the *in vivo* cross sectional morphometric analyses, 1.95 for the interaction term, and 1.55 for *ex vivo* morphometry. However, we should consider that not all animals showed the same learning process and progress, indicating that they may not learn in the same way, or that they process information differently. Thus, future studies including more animals, and a longer task learning time could enable verifying those results and refining the task specificity. Such studies should increase the optimism that useful interventions could be designed for rehabilitation following injury, and for building resilience for neurodegenerative disease. For example, recent efforts have underlined the role of adaptive myelination as a key mechanism for the fine-tuning of neuronal circuitry [50].

### Future directions

*In vivo* imaging of animal models has translational value for human studies of rehabilitation and repair. While most studies to date have used cross-sectional designs, longitudinal studies are necessary for directly estimating the effects of exercise interventions on motor function and cognition over time. Diffusion based imaging adds benefit in terms of increased contrast emphasizing white matter structures, and the ability to detect potential lesions. We believe that rapid *in vivo* diffusion based imaging protocols will provide valuable information on microstructural remodeling. Complementary *ex vivo* studies can add information through complex protocols employing more diffusion directions and higher resolution, and enable validation with histology. These will help increase awareness of methodological limitations, while allowing us to understand at a finer scale the consequences of interventions (in terms of timing, specificity, intensity) on neurons, myelin or astrocytes, and how these interventions could be amended to augment their effects. Furthermore, understanding and fine-tuning plasticity based interventions targeting age-related cognitive decline may offer benefits to broad populations.

## Supporting information

S1 Fig While cortical thicknesses differences did not survive multiple comparison correction, the uncorrected statistical analysis suggested increased cortical thickness in trained animals (reachers). These results pointed to a role for the contralateral motor somatosensory (dysgranular, and forelimb areas) and visual cortices, as well as the ipsilateral piriform cortex (A: coronal cross sections; B: volume/surface rendering. Results are presented as *t*maps, thresholded at 0.05 significance level, using the whole brain as a mask (yellow).
(TIF)
