# Supplementary figures and images for: Magnetic resonance imaging of mouse brain networks plasticity following motor learning
Source: PLoS One. 2019 May 8;14(5):e0216596. doi: 10.1371/journal.pone.0216596 (PMC6505950; doi:10.1371/journal.pone.0216596)

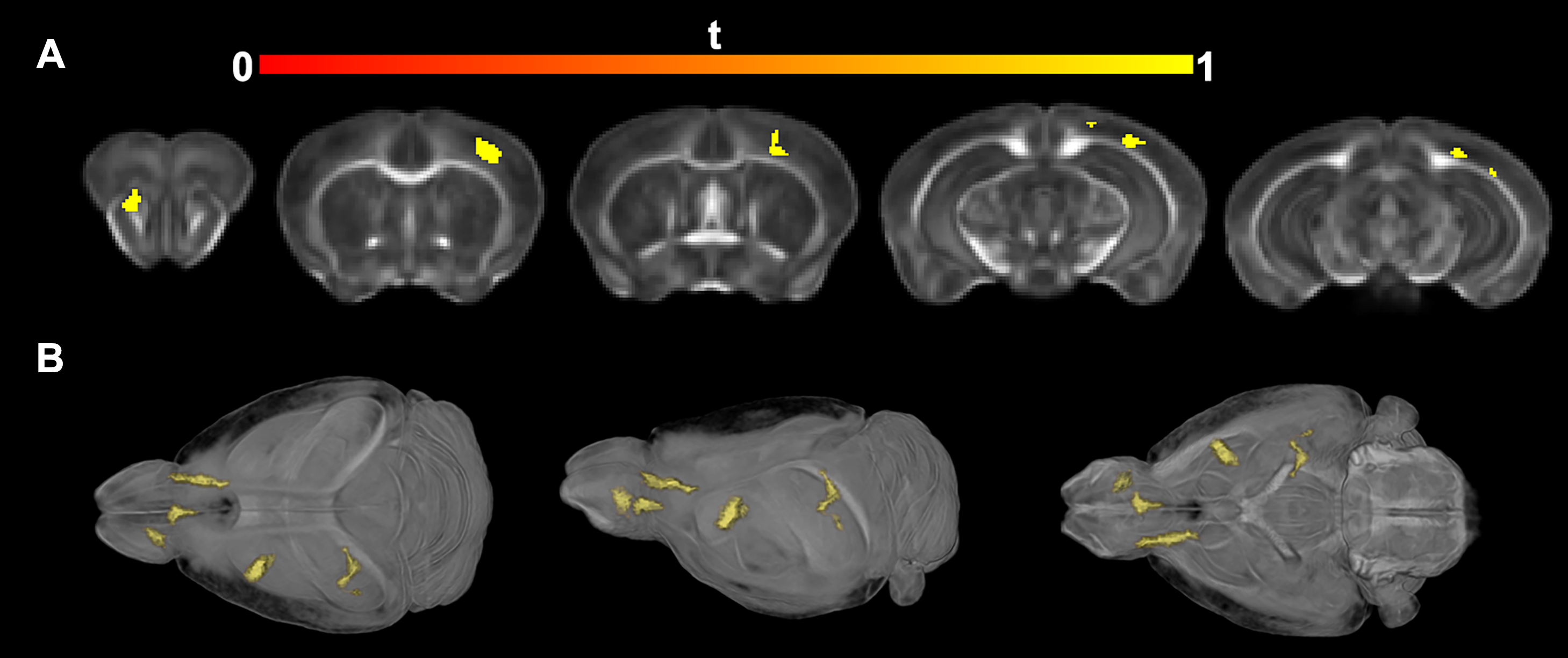

Supplement: S1 Fig — These results pointed to a role for the contralateral motor somatosensory (dysgranular, and forelimb areas) and visual cortices, as well as the ipsilateral piriform cortex (A: coronal cross sections; B: volume/surface rendering. Results are presented as tmaps, thresholded at 0.05 significance level, using the whole brain as a mask (yellow). (TIF) [file pone.0216596.s001.tif]
